# Pancreatic injury in patients treated with immune checkpoint inhibitors: a retrospective multicenterstudy

**DOI:** 10.1007/s00535-024-02083-1

**Published:** 2024-02-29

**Authors:** Kae Nagao, Arata Sakai, Hidetaka Tsumura, Takao Iemoto, Yuichi Hirata, Hitomi Hori, Kyohei Ogisu, Saori Kakuyama, Takuya Ikegawa, Tamaki Hirata, Takeshi Ezaki, Keisuke Furumatsu, Kodai Yamanaka, Takao Kato, Seiji Fujigaki, Hidenori Tanaka, Yosuke Yagi, Takeshi Tanaka, Takashi Kobayashi, Atsuhiro Masuda, Hideyuki Shiomi, Yuzo Kodama

**Affiliations:** 1 Division of Gastroenterology, Department of Internal Medicine, Kobe University Graduate School of Medicine, 7-5-1 Kusunoki-cho, chuo-ku, Kobe, Hyogo 650-0071 Japan; 2 Department of Gastroenterology, Hyogo Cancer Center, Akashi, Hyogo Japan; 3 Department of Gastroenterology, Kita-Harima Medical Center, Ono, Hyogo Japan; 4 Department of Gastroenterology, Kakogawa Central City Hospital, Kakogawa, Hyogo Japan; 5 Department of Gastroenterology, Yodogawa Christian Hospital, Osaka, Osaka Japan; 6 Department of Gastroenterology, Nippon Life Hospital, Osaka, Osaka Japan; 7 Department of Gastroenterology, Takatsuki General Hospital, Takatsuki, Osaka Japan; 8 Department of Gastroenterology, Japanese Red Cross Kobe Hospital, Kobe, Hyogo Japan; 9 Department of Gastroenterology, Nishiwaki Municipal Hospital, Nishiwaki, Hyogo Japan; 10 Department of Gastroenterology, Kobe Medical Center, Kobe, Hyogo Japan; 11 Department of Gastroenterology, Akashi Medical Center, Akashi, Hyogo Japan; 12 Department of Gastroenterology and Hepatology, Osaka Saiseikai Nakatsu Hospital, Osaka, Osaka Japan; 13 Division of Gastroenterology, Konan Medical Center, Kobe, Hyogo Japan; 14 Department of Gastroenterology, Awaji Medical Center, Awaji, Hyogo Japan; 15 Department of Gastroenterology, Hyogo Prefectural Harima-Himeji General Medical Center, Himeji, Hyogo Japan; 16 Department of Gastroenterology, Sanda City Hospital, Sanda, Hyogo Japan; 17 Department of Internal Medicine, Shiso Municipal Hospital, Shiso, Hyogo Japan; 18 Division of Hepatobiliary and Pancreatic Diseases, Department of Gastroenterology, Hyogo Medical University, Nishinomiya, Hyogo Japan

**Keywords:** Pancreatic injury, Pancreatitis, Immune checkpoint inhibitors, Malignant tumors

## Abstract

**Background:**

Immune checkpoint inhibitor-related pancreatic injury (ICI-PI) is a rare occurrence, which has not been reported in detail. We conducted a retrospective multicenter study to determine the clinical characteristics, risk factors, and treatment of ICI-PI.

**Methods:**

We reviewed the medical records of patients who received ICIs for malignant tumors between April 2014 and April 2019 at 16 participating hospitals. Patients with elevated pancreatic enzymes or pancreatitis were identified and classified using the Common terminology Criteria for Adverse Events (CTCAE) ver.5.0). The number of patients with pancreatic enzyme elevation was determined and those with pancreatic enzyme elevation of ≥ grade 3 according to CTCAE ver.5.0, or pancreatitis underwent detailed analysis for ICI-PI.

**Results:**

The study enrolled 1069 patients. Nineteen patients (1.8%) had ICI-PI, 5 (0.5%) of whom also had pancreatitis. Four patients had mild pancreatitis, whereas 1 patient had severe pancreatitis, culminating in death. Steroid therapy was administered to 7 of 19 patients, which led to ICI-PI improvement in 5 patients. On the other hand, ICI-PI improved in 9 of 12 patients who were not administered steroid therapy. Six of the 14 patients with ICI-PI improvement were rechallenged with ICI, and ICI-PI relapse occurred in only 1 patient (16.7%), which improved with ICI discontinuation and steroid therapy.

**Conclusions:**

ICI-PI is a rare occurrence, with a low incidence of pancreatitis, which followed a very serious course in one patient. Although the benefit of steroid therapy for ICI-PI is unclear, ICI rechallenge is acceptable after improvement of ICI-PI without pancreatitis.

**Supplementary Information:**

The online version contains supplementary material available at 10.1007/s00535-024-02083-1.

## Introduction

Immune checkpoint inhibitors (ICIs) have been approved as standard therapy for several malignant tumors [1–4]. Associations between ICI efficacy and predictors of treatment response such as programmed cell death ligand 1 (PD-L1), tumor mutational burden (TMB), and microsatellite instability/defective mismatch repair (MSI/dMMR) have been reported [5–10]. Already, not only the characteristics of suitable patients for ICI has been further clarified, but also the indications for ICI are being expanded across the cancer spectrum.

On the other hand, the administration of ICIs is associated with immune-related adverse events (irAEs), which commonly involve the dermatologic, gastrointestinal, hepatic, pulmonary, and endocrine systems [11, 12] and their management has been established [13, 14]. However, pancreatic injuries are rare irAE manifestations of ICI administration [15–20], and their incidence, risk factors, treatments, clinical course, and associated OS are unclear. The National Comprehensive Cancer Network (NCCN) guidelines recommend continuation of ICI therapy in asymptomatic patients with pancreatic enzyme elevation with normal findings on pancreatic imaging [21]. The NCCN guidelines also recommend discontinuation of ICI therapy and administration of steroids for patients with symptomatic pancreatitis [21]. However, these guidelines are based on weak evidence, necessitating large-scale studies focusing on pancreatic injuries after ICI administration. Therefore, we conducted a retrospective multicenter study to determine the clinical characteristics, risk factors, and treatment of ICI-related pancreatic injury (ICI-PI).

## Methods

### Study design and population

This multicenter retrospective study was conducted at 16 hospitals. All patients who received ICI therapy for malignant tumors between April 2014 and March 2019 at the participating hospitals were included. After a medical chart review, patients who did not undergo measurement of pancreatic enzymes during the study period were excluded from the analysis.

The study protocol was approved by Kobe University’s Clinical Research Ethical Committee (No. B200343) and the corresponding body at each participating hospital. The requirement of informed consent was waived because of the retrospective study design. Information about the study was disclosed on each hospital’s website, providing eligible patients with an opportunity to opt out of the analysis. This study was conducted in accordance with the Declaration of Helsinki. All authors had access to the study data, and reviewed and approved the final manuscript. Tumor stage at diagnosis or ICI administration was classified according to the version of the Union for International Cancer Control (UICC) system in use at that time [22].

### Outcomes and definitions

The primary endpoint was the incidence of ICI-PI. The secondary endpoints included the incidence of ICI-related pancreatitis, risk factors for ICI-PI, treatments for ICI-PI and OS.

ICI-PI was defined as ≥ grade 3 elevation of the serum pancreatic enzymes (amylase or lipase) after initiation of ICI therapy, based on the modified Common terminology Criteria for Adverse Events (CTCAE) ver.5.0 (Table S1) [23], in the absence of any other obvious etiology such as pancreatic tumor or pancreatic stones. This definition aligned with that employed by the most recent study that focused on ≥ grade 3 pancreatic enzyme elevations classified according to CTCAE ver.4.0 [16, 17]. Patients were diagnosed with ICI-PI with pancreatitis if they satisfied two or more of the following conditions: (1) had ICI-PI according to the above-mentioned definition, (2) experienced abdominal pain with no cause other than pancreatitis, and (3) showed imaging findings of pancreatitis. The imaging findings of ICI-related pancreatitis were classified into the acute pancreatitis-like (AP-like) and autoimmune pancreatitis-like (AIP-like) categories, according to Das et al.’s study [24]. The AP-like features were considered present if the imaging findings met the Atlanta criteria: presence of (1) pancreatic enlargement (focal or diffuse) (2) heterogenous parenchymal enhancement (focal or diffuse) (3) peripancreatic stranding, (4) acute peripancreatic fluid collection, (5) acute necrotic collections, (6) pancreatic necrosis, (7) peripancreatic necrosis, and (8) main pancreatic duct dilatation [25]. The AIP-like imaging features were assessed per the HISORt criteria as follows: (1) diffuse gland enlargement with rim enhancement, (2) diffuse/irregular attenuation of the main pancreatic duct, (3) focal pancreatic enlargement, (4) focal pancreatic duct stricture, (5) pancreatic atrophy, and (6) pancreatic calcification [26]. Atypical imaging features that did not fall into the AP-like or AIP-like category were designated as “other.” The severity of ICI-PI with pancreatitis was classified according to CTCAE ver.5.0 (Table S1) [23]. Improvement of ICI-PI was defined as amelioration of pancreatic enzyme elevation to grade 1 (CTCAE ver.5.0) [23].

OS was defined as the time interval between initiation of ICI therapy and death due to any cause, and the data of patients were censored if they were not dead.

### Data analysis and statistics

All statistical analyses were performed using SPSS version 28 (IBM, Armonk, NY). For univariate analysis, continuous or ordinal variables (such as age) were described as the median (range) and compared using the Mann–Whitney *U* test, while categorical variables (such as sex) were presented as the number of cases and proportions and compared using Fisher’s exact test. OS was evaluated using Kaplan–Meier curves and log-rank tests. All statistical tests were two-tailed, and statistical significance was set at *P* < 0.05.

## Results

### Patient characteristics

**Fig. 1:**
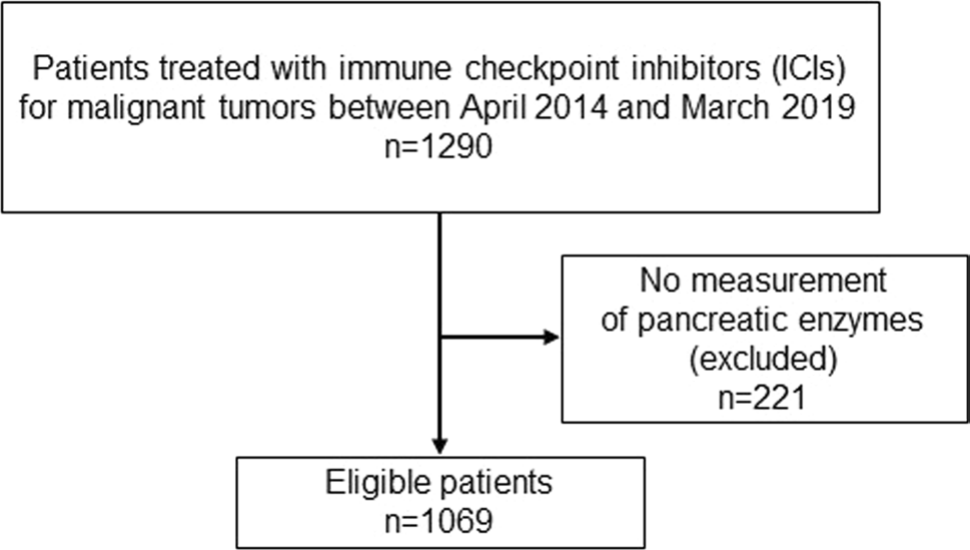
Flowchart of eligible patients. This study initially enrolled 1290 patients. Of these, 221 patients whose pancreatic enzymes were not measured during the study period were excluded, and finally 1069 patients were considered eligible for inclusion in the study

Initially, 1290 patients were enrolled in this retrospective analysis; 221 patients who did not undergo pancreatic enzyme measurement during the study period were excluded, Finally, 1069 patients were eligible for study inclusion (Fig. 1). Table S2 provides information on the participating hospitals and the number of eligible patients. The patient characteristics are presented in Table 1. The study population predominantly comprised men, and lung cancer (60.4%) was the most common primary disease. At the time of ICI administration, 68.7% of patients had stage IV disease, followed by stage III disease in 15.3% and postoperative cancer recurrence in 14.7% of patients. Most patients were treated with programmed cell death (PD)-1 inhibitors (91.4%), followed by PD-L1 inhibitors (9.4%), and cytotoxic T lymphocyte antigen (CTLA)-4 inhibitors (1.6%). Multiple ICI monotherapy regimens (with different drugs) were administered to 1.9% of patients and combination therapy with different ICIs was administered to 0.9% of patients. 762 patients had prior use of cytotoxic anticancer drugs, of which 707 (92.8%), the majority of patients, had prior use of multiple cytotoxic anticancer drugs. 32 (3.0%) patients had prior use of interferon (IFN) therapy. 427 patients had prior use of molecular targeted drugs, of these, 132 (30.9%) patients were on multiple monotherapy regimens and 8 (1.9%) patients were on combination regimens of multiple drugs. There was no concomitant use of ICI and cytotoxic anticancer or molecular targeted drugs at the same time.

**Table 1:** Patients’characteristics

n = 1069	
Median age, years (range)	69 (25–91)
Sex, *n* (%)	
Male	818 (76.5)
Female	251 (23.5)
Median BMI (range)	21.1 (10.23–39.27)
Diabetes, *n* (%)	
Present	205 (19.2)
Absent	864 (80.8)
Alcohol intake, *n* (%)	
≥ 50 g/day	189 (17.7)
< 50 g/day	880 (83.3)
Smoking, n (%)	
Present (former or current)	764 (71.5)
Absent (never)	305 (28.5)
Allergy, n (%)	
Present	269 (25.2)
Absent	800 (74.8)
History of autoimmune disease, n (%)	
Present	32 (3.0)
Absent	1037 (97.0)
History of pancreatitis, n (%)	
Present	6 (0.6)
Absent	1063 (99.4)
Family history of cancer, n (%)	
Present	172 (16.1)
Absent	897 (83.9)
Primary disease, n (%)	
Lung cancer	646 (60.4)
Gastric cancer	152 (14.2)
Renal cancer	123 (11.5)
Melanoma	57 (5.3)
Ureteral/bladder cancer	53 (5.0)
Head and neck cancer	23 (2.1)
Others	15 (1.4)
Stage at initial diagnosis (UICC), n (%)	
I	66 (6.1)
II	84 (7.8)
III	311 (29.1)
IV	597 (55.8)
Unknown	3 (0.3)
Stage at start of ICI treatment (UICC), n (%)	
I	3 (0.3)
II	11 (1.0)
III	164 (15.3)
IV	734 (68.7)
Postoperative cancer recurrence	157 (14.7)
Pancreatic metastasis, n (%)	
Present	27 (2.5)
Absent	1042 (97.5)
Type of ICI treatment, n (%)	
PD-1 inhibitor	977 (91.4)
PD-L1 inhibitor	101 (9.4)
CTLA-4 inhibitor	17 (1.6)
Multiple ICI treatment, n (%)	
Multiple ICI monotherapy	20 (1.9)
ICI Combination therapy	10 (0.9)
Median number of ICI treatments, times (range)	6 (1–120)
Median duration of ICI treatment, months (range)	3.3 (0–67)
History of cytotoxic chemotherapy, n (%)	
Present	762 (71.3)
Absent	307 (28.7)
History of IFN therapy, n (%)	
Present	32 (3.0)
Absent	1037 (97.0)
History of molecular targeted drugs, n (%)	
Present	427 (40.0)
Absent	642 (60.0)
Other organ disorders, n (%)	
Present	324 (30.3)
Absent	745 (69.7)
Liver disorder	77 (7.2)
Gastrointestinal disorder	33 (3.1)
Endocrine disorder	112 (10.5)
Lung disorder	68 (6.4)
Skin disorder	53 (5.0)
Median observation period, months (range)	12.3 (0.1–77.3)
Median overall survival, months (range)	15.8 (13.9–17.8)

*BMI* body mass index, *UICC* Union for International Cancer Control, *ICI* immune checkpoint inhibitor, *PD-1* programmed cell death 1, *PD-L1* programmed cell death-ligand 1, *CTLA-4* cytotoxic T-lymphocyte-associated protein 4, *multiple ICI monotherapy* use of different ICIs as monotherapy, *ICI combination therapy* use of two or more ICIs together at the same time

Other organ disorders considered to be irAEs were observed in 324 (30.3%) patients. The most common type of irAE is endocrine disorders such as thyroid and adrenal dysfunction, followed by liver disorders, gastrointestinal disorders, pneumonia, and dermatitis.

The median number of ICI treatments was 6, and the median duration of ICI treatment was 3.3 months. The median observational period was 12.3 months and the median OS was 15.8 months.

### Incidence of ICI-PI and pancreatitis

The incidences of pancreatic enzyme elevation and ICI -related pancreatic injury are shown in Table 2. Four grades of pancreatic enzyme elevation were observed in 160 (15%) of 1069 patients. The primary endpoint, i.e., ICI-PI, was present in 19 (1.8%) patients, of which 18 (1.7%) patients had grade 3 ICI-PI and 1 (0.1%) patient had grade 4 ICI-PI. Five (0.5%) patients developed pancreatitis: 4 patients had grade 2 pancreatitis, and 1 patient had severe pancreatitis culminating in death (grade 5). Except for the patient with severe pancreatitis, all patients with ICI-PI were asymptomatic.

**Table 2:** Incidence of pancreatic enzyme elevation, ICI-related pancreatic injury, and pancreatitis

Total	*n* = 1069
All grades of pancreatic enzyme elevation, *n* (%)	160 (15)
Grade 1	93 (8.7)
Grade 2	48 (4.5)
Grade 3	18 (1.7)
Grade 4	1 (0.1)
ICI-related pancreatic injury (ICI-PI), *n* (%)	19 (1.8)
ICI-PI without pancreatitis, *n* (%)	14 (1.3)
ICI-PI with pancreatitis, *n* (%)	5 (0.5)
Grade 2	4 (0.4)
Grade 3	0 (0)
Grade 4	0 (0)
Grade 5	1 (0.1)

*ICI* immune checkpoint inhibitor, *CTCAE* Common Terminology Criteria for Adverse Events, *grade* grade of pancreatic enzyme elevation or pancreatitis (Citation modified from CTCAEver.5.0), *ICI-PI* ICI-related pancreatic injury (≥ grade 3 of pancreatic enzyme elevation)

### Risk of ICI-PI and OS

Table 3 shows the results of the univariate analysis investigating the risk factors related to ICI-PI. Renal cancer (OR 7.33, 95% CI 2.90–18.04, *p* < 0.001), malignant melanoma (OR 4.96, 95% CI 1.59–15.51, *p* = 0.015), CTLA-4 inhibitors (OR 21.27, 95% CI 6.21–72.86, *p* < 0.001), multiple ICI monotherapy (OR 6.75, 95% CI 1.45–31.38, *p* = 0.047), combination ICI therapy (OR 27.94, 95% CI 6.62–117.90, *p* < 0.001), past history of IFN therapy (OR 13.53, 95% CI 4.55–40.26, *p* < 0.001), and complications such as irAEs in other organs (OR 9.00, 95% CI 2.96–27.31, *p* < 0.001), especially in the liver (OR 6.36, 95% CI 2.35–17.25, *p* < 0.001) and endocrine system (OR 6.62, 95% CI 2.60–16.82, *p* = 0.001), were associated with a significantly higher risk of ICI-PI. Of the 15 ICI-PI patients with irAEs in other organs, ICI-PI preceded the other irAEs in 2 (13%) patients, ICI-PI occurred concomitantly with other irAEs in 3 (20%) patients, and ICI-PI occurred after other irAEs in 10 (67%) patients. The other irAEs associated with ICI-PI were endocrine disorders in 8 (42.1%) cases, liver disorders in 6 (31.6%) cases, gastrointestinal disorders in 2 (10.5%) cases, and pneumonia and dermatitis in 1 (5.3%) case.

**Table 3:** Risk analysis of ICI-related pancreatic injury

	Total *n* = 1069	ICI-related pancreatic injury present *n* = 19 (1.8%)		ICI-related pancreatic injury absent *n* = 1050 (98.2%)	Odds ratio	(95%CI) *p*-value
Median age, years (range)		67 (57–78)	69 (25–91)		–	0.60
Sex, *n* (%)						
Male	818	15 (78.9)	803 (76.5)		0.87 (0.29–2.64)	1.00
Female	251	4 (21.1)	247 (23.5)			
Median BMI (range)		21.2 (17.8–24.8)	21.1 (10.2–39.3)		–	0.70
Diabetes, *n* (%)						
Present	205	3 (15.8)	202 (19.2)		0.79 (0.23–2.73)	1.00
Absent	864	16 (84.2)	848 (80.8)			
Alcohol intake, *n* (%)						
≥ 50 g/day	189	4 (21.1)	185 (17.6)		1.25 (0.41–3.81)	0.76
< 50 g/day	880	15 (78.9)	865 (82.4)			
Smoking, *n* (%)						
Present	764	16 (84.2)	748 (71.2)		2.15 (0.62–7.44)	0.31
Absent	305	3 (15.8)	302 (28.8)			
History of autoimmune disease, *n* (%)						
Present	32	0 (0)	32 (3.0)		–	1.00
Absent	1037	19 (100)	1018 (97.0)			
History of pancreatitis, *n* (%)						
Present	6	0 (0)	6 (0.6)		–	1.00
Absent	1063	19 (100)	1044 (99.4)			
Primary disease, *n* (%)						
Lung cancer	646	4 (21.1)	642 (61.1)		0.17 (0.056–0.52)	< 0.001*
Gastric cancer	152	1 (5.3)	151 (14.4)		0.33 (0.04–2.50)	0.50
Renal cancer	123	9 (47.4)	114 (10.9)		7.33 (2.90–18.04)	< 0.001*
Malignant melanoma	57	4 (21.1)	53 (5.0)		4.96 (1.59–15.51)	0.015*
Ureteral/bladder cancer	53	1 (5.3)	52 (5.0)		1.06 (0.14–8.10)	1.00
Head and neck cancer	23	0 (0)	23 (2.2)		–	1.00
Others	15	0 (0)	15 (1.4)		–	1.00
Type of ICI used, *n* (%)						
PD-1 inhibitor	977	17 (89.5)	960 (91.4)		0.80 (0.18–3.50)	0.68
PD-L1 inhibitor	101	2 (10.5)	99 (9.4)		1.13 (0.26–4.96)	0.70
CTLA-4 inhibitor	17	4 (21.1)	13 (1.2)		21.27 (6.21–72.86)	< 0.001*
Multiple ICI treatment, *n* (%)						
Multiple ICI monotherapy	20	2 (10.5)	18 (1.7)		6.75 (1.45–31.38)	0.047*
ICI combination therapy	10	3 (15.7)	7 (0.6)		27.94 (6.62–117.90)	< 0.001*
Median number of ICI treatments, times (range)		4 (2–60)	6 (1–120)		–	0.57
History of cytotoxic chemotherapy, *n* (%)						
Present	762	6 (31.6)	756 (72.0)		0.18 (0.068–0.49)	< 0.001*
Absent	307	13 (68.4)	294 (28.0)			
History of IFN therapy, *n* (%)						
Present	32	5 (26.3)	27 (2.6)		13.53 (4.55–40.26)	< 0.001*
Absent	1037	14 (73.7)	1023 (97.4)			
History of molecular targeted drugs, *n* (%)						
Present	427	8 (42.1)	419 (40.0)		1.10 (0.44–2.75)	0.82
Absent	642	11 (57.9)	631 (60.0)			
Other organ disorders, *n* (%)						
Present	324	15 (78.9)	309 (29.4)		9.00 (2.96–27.31)	< 0.001*
Absent	745	4 (21.1)	741 (70.5)* F1*			
Liver disorder	77	6 (31.6)	13 (1.2)		6.36 (2.35–17.25)	< 0.001*
Gastrointestinal disorder	33	2 (10.5)	13 (1.2)		3.87 (0.86–17.47)	0.11
Endocrine disorder	112	8 (42.1)	11 (1.0)		6.62 (2.60–16.82)	0.001*
Lung disorder	68	1 (5.3)	18 (1.7)		0.82 (0.11–6.20)	1.00
Skin disorder	53	1 (5.3)	18 (1.7)		1.07 (0.14–8.14)	1.00
Median OS, months (95% CI)		21.8 (5.5–38.0)	15.8 (13.4–17.7)		–	0.41

*ICI* immune checkpoint inhibitor, *95%CI* confidence interval, *BMI* body mass index, *UICC* Union for International Cancer Control, *PD-1* programmed cell death 1, *PD-L1* programmed cell death-ligand 1, *CTLA-4* cytotoxic T-lymphocyte-associated protein 4, *multiple ICI monotherapy* use of different ICIs as monotherapy, *ICI Combination therapy* use of two or more ICIs together at the same time, *IFN* interferon,*OS* overall survival

**Fig. 2:**
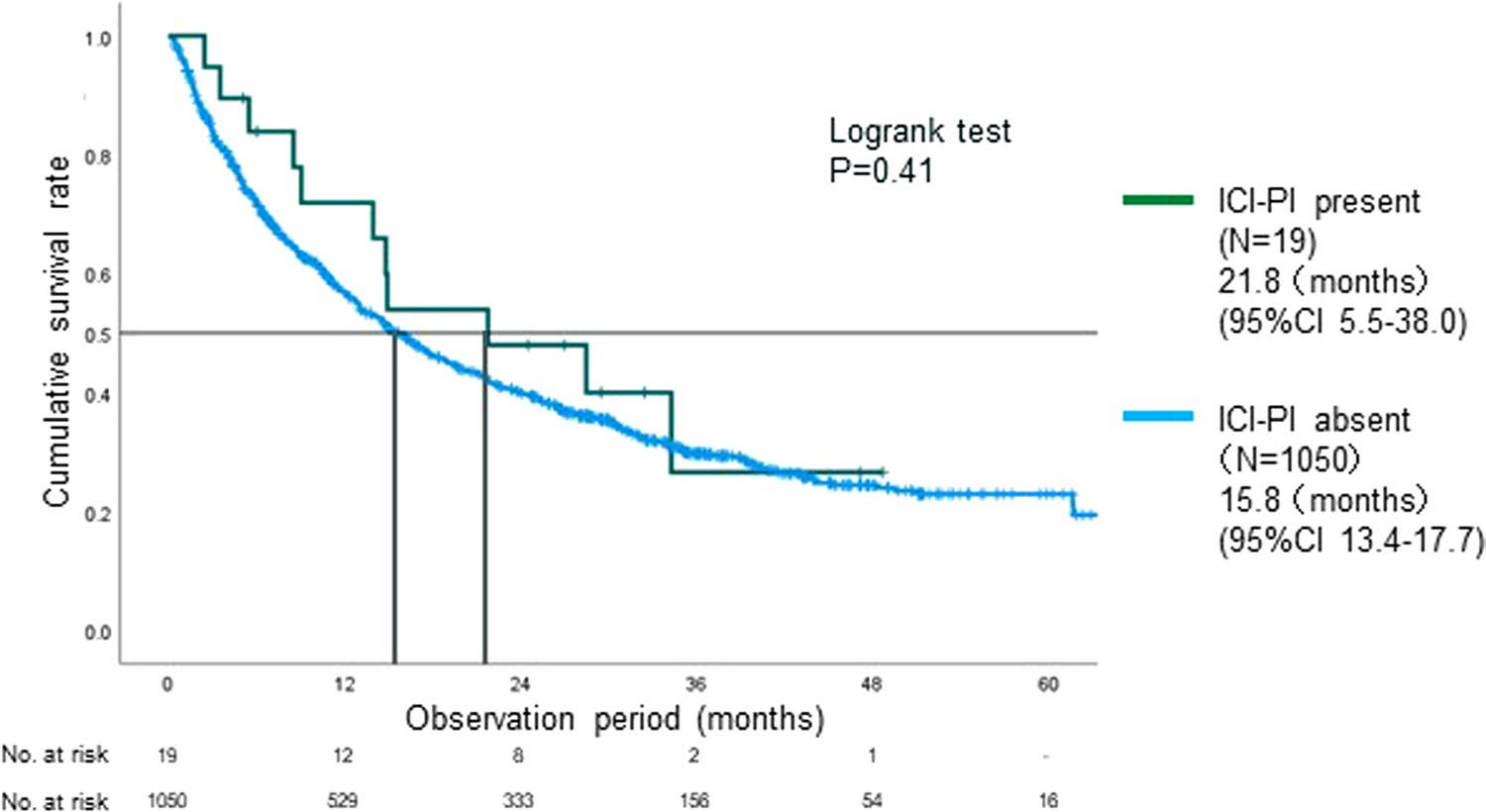
Kaplan–Meier curve stratified by the presence or absence of ICI-related pancreatic injury (ICI-PI). There was no significant difference in the overall survival between patients with and without ICI-PI

On the other hand, the risk of ICI-PI was significantly lower for lung cancer (OR 0.17, 95% CI 0.056–0.52, *p* < 0.001) and prior treatment with cytotoxic anticancer agents (OR 0.18, 95% CI 0.068–0.49, *p* < 0.001). The participants’ OS is shown in Table 3, while Fig. 2 depicts the Kaplan–Meier curve stratified by the presence or absence of ICI-PI. There was no significant difference in the OS of patients with and without ICI-PI (21.8 months, 95% CI 5.5–38.0 vs 15.8 months, 95% CI 13.4–17.7, *p* = 0.41).

### Clinical course of ICI-PI

**Fig. 3:**
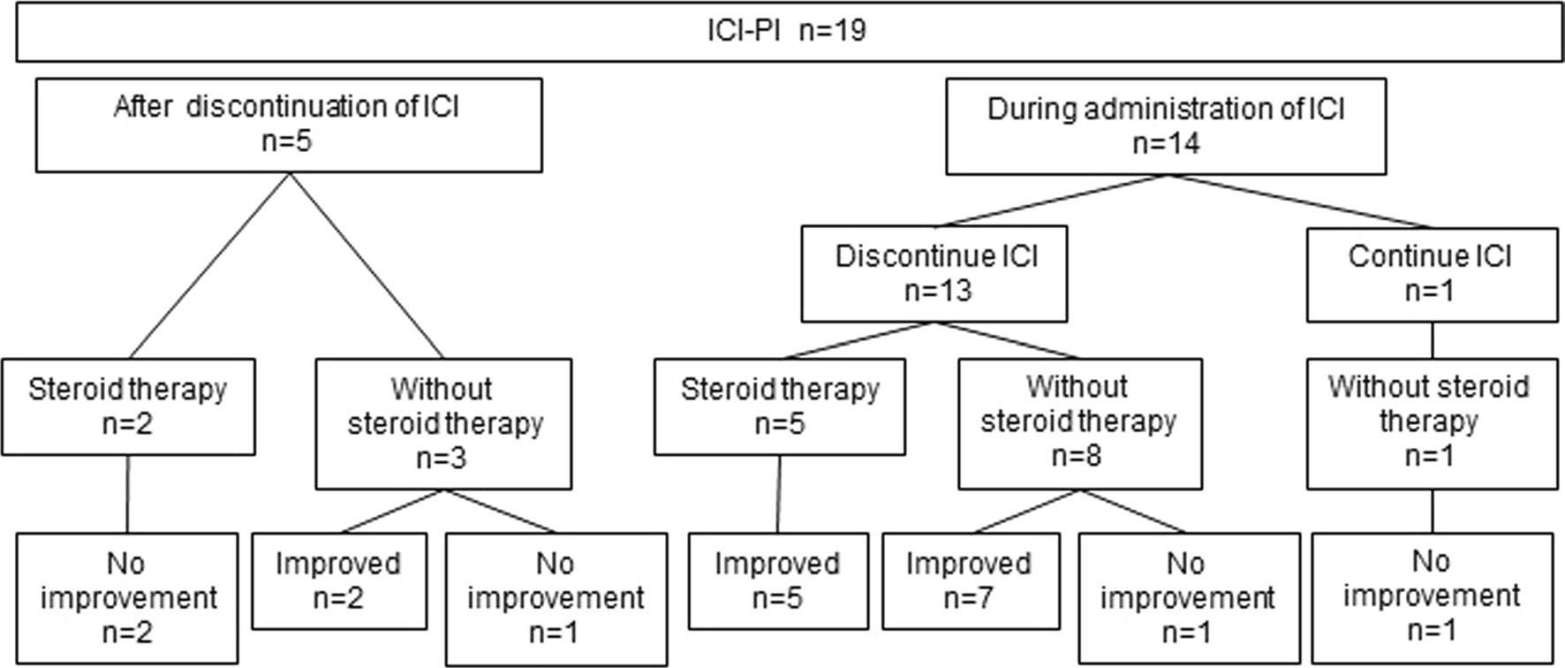
Clinical course of patients with ICI-related pancreatic injury (ICI-PI). Five of 19 patients developed ICI-PI after discontinuation of ICI and 14 developed it during administration of ICI. ICI was discontinued in 13 of the 14 patients, and 1 patient continued ICI therapy. Steroid therapy was administered to 7 of 19 patients, and ICI-PI improved in 5 patients. Twelve patients were not administered steroid therapy, and ICI-PI improved in 9 of them

The clinical course of patients with ICI-PI is shown in Fig. 3. The median time from commencement of ICI therapy to the onset of ICI-PI was 92 days (19–706). Five of 19 patients developed ICI-PI after discontinuation of ICI (2 weeks, 1 month, 1 month, 1 month, 5 months,) and 14 developed ICI-PI during ICI administration. ICI was discontinued because of ICI-PI in 13 of the 14 patients. One patient continued ICI for 6 weeks, which was subsequently discontinued due to pneumonia. Steroid therapy was administered to 7 of 19 patients, and ICI-PI improved in 5 patients. On the other hand, 12 patients were not administered steroid therapy, and ICI-PI improved in 9 of these patients.

**Fig. 4:**
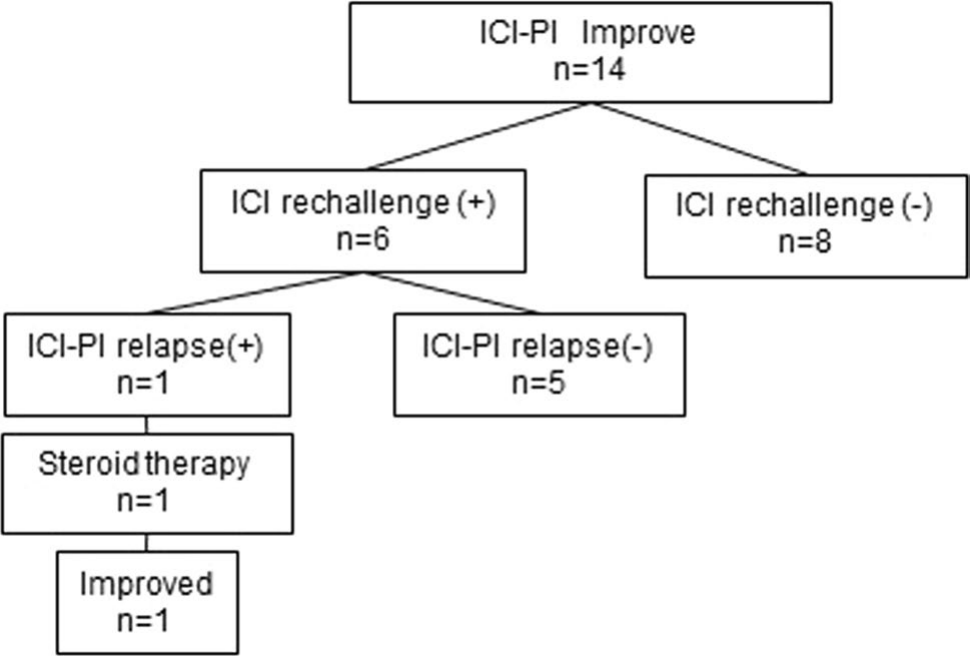
Rechallenge with ICI after improvement in ICI-PI. Six of 14 patients with ICI-PI improvement faced the ICI rechallenge: 5 of 6 patients were rechallenged with the same ICI, and 1 patient received another ICI. ICI-PI relapse was observed in only 1 patient, which improved with ICI discontinuation and steroid therapy

Six of the 14 patients with ICI-PI improvement were rechallenged with ICIs. All six cases were Grade 3 ICI-PI without pancreatitis. Five of 6 patients were rechallenged with the same ICI (PD-1 inhibitor), and while 1 patient changed ICI from CTLA-4 inhibitor to PD-L1 inhibitor. ICI-PI relapse was observed in only 1 patient (16.7%), which improved with ICI discontinuation and steroid therapy (Fig. 4).

Table S3 summarizes the cases of 5 patients with ICI-PI with pancreatitis [27, 28]. Two of 5 patients developed ICI-PI after discontinuation of ICI (1.5 months, 3 months) and 3 development pancreatic injury during ICI treatment. One of the 3 patients (No. 4) had a recurrence of asymptomatic pancreatic enzyme elevation during rechallenge with the same ICI and new pancreatitis findings on CT. Regarding CT findings, 1 showed severe AP-like findings, 3 had AIP-like findings, and 1 was classified as “other” (pancreatic atrophy).

ICI was discontinued because of ICI-PI in all patients. Steroid therapy was administered in 4 of the 5 cases. Two patients (No.4,5) started steroids when the findings of pancreatitis were discovered, 1 patient (No.3) started for pituitaritis occurring at the same period. One patient (No.1) started them 2 months later, at the time he developed colitis. As for outcome, 4 patients had improved ICI-PI with pancreatitis. One patient (No.5) with a history of nivolumab use and prior liver injury, who had asymptomatic elevated pancreatic enzymes, was admitted 2 days later with severe pancreatitis with abdominal pain and died 3 days later despite steroid and fluid infusion therapy [28]. Since the patient had a history of both nivolumab and pazopanib use, a pathological autopsy was performed to determine the cause of death. the gross specimen of pancreas showed parenchymal and fat necrosis with bleeding, and Inflammation had spread to the retroperitoneum, abdominal cavity, transverse colon, and left adrenal gland. Microscopically, the fat necrosis was observed around the parenchymal necrosis. Inflammatory cell infiltration centering on neutrophils was observed in necrotic lesions of the pancreatic parenchyma. In the remaining pancreatic parenchyma, In addition, markedly more CD8-positive T cells were detected than CD4-positive T cells. The result of pathological autopsy was suggestive of ICI-PI, but the possibility of pancreatitis caused by pazopanib could not be eliminated. Because of previous liver injury, the liver was markedly infiltrated with inflammatory cells in the portal region and parenchyma, and a CD8-predominant T lymphocyte infiltrate was also present. There were no findings suspicious of cardiac irAE or other causes of the acute course of the disease. The cause of death was thought to be multiple organ failure, mainly due to acute pancreatitis.

## Discussion

This large-scale multicenter study investigated the incidence, risk factors, and clinical course of ICI-PI in Japan. The results showed that ICI-PI is a rare phenomenon, with an incidence of 1.8%. Pancreatitis was even rarer, with an incidence of 0.5%, but one patient succumbed to severe pancreatitis. Previous studies have included various ethnic groups, but most patients in this study were Japanese. This is the first study to identify the risk factors for ICI-PI in an Asian population.

Although the reported incidence of ICI-PI differs among studies, a recent meta-analysis found that asymptomatic lipase elevation occurs in 2.7% of patients after ICI use [16]. The incidence of ICI-PI with pancreatitis has been reported to be approximately 0.3–3.9% [15, 16, 29–31]. The largest single-center retrospective study to date reported that grade 3 (CTCAE ver.4.0) or higher lipase elevations were seen in 4% and pancreatitis in 1.4% of patients who received ICI [17]. Our study classified ICI-PI as ≥ grade 3 elevation in the serum pancreatic enzymes (amylase or lipase) after initiation of ICI according to CTCAE ver.5.0, which was observed in 1.8% of patients. Previous studies classified pancreatic enzyme elevation according to CTCAE ver.4.0; therefore, upon reclassification of our results according to the CTCAE ver.4.0, the frequency of ICI-PI rose to 4.0%, consistent with the findings of previous studies. ICI-related pancreatitis in our study was even rarer with an incidence of 0.5%, which was also similar to the findings of previous studies.

According to the study of Das JP, et al., the most common CT findings of pancreatitis were AP-like (80%), AIP-like (16%), and a mixed pattern (4%) [24]. A variety of imaging findings were observed in our study, since one of the 5 patients with pancreatitis showed severe AP-like findings, 3 patients showed AIP-like findings, and 1 patient was classified as “other” (pancreatic atrophy only). Patient No.3 in table S3, who showed AIP-like imaging findings, was described in detail in a previous study by Tanaka et al. Magnetic resonance imaging showed diffuse enlargement and focal restricted diffusion, endoscopic ultrasound showed diffuse hypoechoic enlargement with hyperechoic foci or stranding, and endoscopic retrograde pancreatography showed skipped narrowing of the main pancreatic duct [27]. Researchers have suggested that the suppression of immunomodulation by ICI affects the pancreas, resulting in imaging features that resemble autoimmune pancreatitis.

ICI-related pancreatitis is reportedly mild in most cases and follows a favorable clinical course [16, 27]. However, we encountered 1 patient with severe pancreatitis culminating in death, who had a history of both ICI and molecular targeted drug (pazopanib) therapy [28]. The autopsy findings suggested that, although the influence of pazopanib cannot be ruled out, it is possible that the pancreatitis was a manifestation of an irAE. Expansion of the indications and application of ICI for various cancers is expected, and the use of combined therapy with various anticancer drugs, including molecular targeted therapy, is expected to increase in the near future, which may lead to the rise in the incidence of ICI-related pancreatitis.

A previous study found no demonstrable superiority of ICI interruption, fluid infusions, or steroids as treatments for ICI-PI, although fluid infusions were associated with a decreased risk of long-term adverse events such as chronic pancreatitis and diabetes [17]. In our study, due to the small number of patients with ICI-PI, especially those with ICI continuation, it was not possible to evaluate the effects of ICI discontinuation, fluid infusions, and steroids on ICI-PI. However, none of the patients with ICI-PI without pancreatitis experienced relapse of ICI-PI when rechallenged with ICI. It is considered acceptable to continue or rechallenge patients with ICI, while closely monitoring the patient for disease progression and further adverse events. On the other hand, no patient faced an ICI rechallenge after the onset of pancreatitis in this study. Although the NCCN guidelines suggest considering ICI rechallenge after improvement of pancreatic injury in the absence of severe pancreatitis [21], it seems appropriate to approach ICI rechallenge with caution since further severe pancreatitis can be fatal.

Some studies have reported on the risk factors for ICI-PI [16, 17, 19, 32]. George et al. reported that CTLA-4 inhibitors, combination therapy, and malignant melanoma were high-risk factors for ICI-PI [21]. The ICI-PI risk factor analysis in the present study incorporated several parameters that were not considered in previous studies, including non-lung cancer, renal cancer, negative history of cytotoxic chemotherapy, history of IFN therapy, and complications with disorders of other organ systems. Interestingly, IFN therapy is involved in the activation of T-cell-based immunity, which may facilitate autoimmune-like reactions.

This study has some limitations. First, the conditions for measuring pancreatic enzymes were not standardized owing to the retrospective-observational study design. It is possible that the diagnosis of ICI-PI was missed in some cases. In addition, patients without pancreatic enzyme measurements were excluded from this study, which could have included asymptomatic ICI-PI. Second, the potential effect of other factors affecting pancreatic injury, such as drug-induced pancreatitis caused by other medications besides ICI, cannot be eliminated completely. Although we excluded patients when other drugs were clearly determined to be the cause of pancreatic injury, one of the five patients with pancreatitis had a history of pazopanib treatment. We did not exclude this patient because ICI-PI and irAEs in other organs can develop after discontinuation of ICI [33]. Third, there is a possibility of confounding in the ICI-PI risk factors that showed statistically significant differences. Although it would have been desirable to perform a multivariate analysis, this was not possible due to the small number of ICI-PI patients. For example, several patients with renal cancer and malignant melanoma who showed statistically significant differences in the univariate analysis received IFN as postoperative treatment, and CTLA-4 inhibitors and ICI combination therapy were administered more frequently to patients with malignant melanoma. ICIs were often administered in the late phase of lung cancer, and there was a history of administration of cytotoxic anticancer agents such as platinum drugs. It is possible that the administration of ICIs to patients with an immunocompromised status due to cytotoxic anticancer drug administration may have reduced the incidence of autoimmune-like reactions. Despite these limitations, to the best of our knowledge, this is the first single large-scale study to report both the incidence and risk factors of ICI-PI.

In summary, ICI-PI was a rare occurrence, and a small subset of patients had pancreatitis, which proved fatal in 1 patient. Although the benefit of steroid therapy and fluid infusions for ICI-PI could not be clarified, ICI rechallenge is acceptable after improvement of ICI-PI without pancreatitis. Although several aspects of ICI-PI remain unelucidated, further studies are needed to explore the pathogenesis and appropriate management methods of ICI-PI.

### Supplementary Information

Below is the link to the electronic supplementary material.

Supplementary file1 (PDF 168 KB)
