# Supplementary material for: Pancreatic injury in patients treated with immune checkpoint inhibitors: a retrospective multicenterstudy
Source: J Gastroenterol. 2024 Feb 29;59(5):424–33. doi: 10.1007/s00535-024-02083-1 (PMC11033227; doi:10.1007/s00535-024-02083-1)
Supplement: Supplementary file 1 — Supplementary file1 (PDF 168 KB) [file 535_2024_2083_MOESM1_ESM.pdf]

Supplementary Table 1: Grade of pancreatic enzyme elevation and pancreatitis {Citation modified from CTCAE ver.5.0 [23]}

	Grade 1	Grade 2	Grade 3	Grade 4	Grade 5
Pancreatic enzyme elevation	>ULN – 1.5 x ULN	>1.5 – 2.0 x ULN; 2.0 – 5.0 x ULN and asymptomatic	>2.0 – 5.0 x ULN with signs or symptoms; >5.0 x ULN and asymptomatic	>5.0 x ULN and with signs or symptoms	-
Pancreatitis	-	Enzyme elevation; radiologic findings only	Severe pain; vomiting; medical intervention indicated (e.g., analgesia, nutritional support	Life-threatening consequences; urgent intervention indicated	Death

CTCAE, Common Terminology Criteria for Adverse Events; ULM, Upper Limit of Normal

Supplementary Table 2: Participating hospitals and number of patients

Institution	Number of patients
Hyogo Cancer Center	350
Kobe University Hospital	208
Kita-harima Medical Center	103
Kakogawa Central City Hospital	97
Yodogawa Christian Hospital	85
Nippon Life Hospital	57
Takatsuki General Hospital	46
Kobe Red Cross Hospital	38
Nishiwaki Municipal Hospital	22
Kobe Medical Center	18
Akashi Medical Center	17
Konan Medical Center	10
Awaji Medical Center	7
Steel Memorial Hirohata Hospital	5
Sanda City Hospital	4
Shiso Municipal Hospital	2
Total	1069

Supplementary Table 3: Clinical course of patients with ICI-PI with pancreatitis

	Sex	Age	Primary disease	ICI	Grade	Duration from pancreatic enzyme elevation	Other organ disorders	CT findings	Discontinuation of ICI	Steroid therapy	Timing of steroid therapy from pancreatitis	Fluid infusion therapy	Outcome	Rechallenge of ICI
1	F	70	Ureteral cancer	nivolumab	2	same time	Endocrine Skin Gastrointestinal (colitis)	AIP-like	Discontinued	+ PSL 30mg/day (colitis)	2months	+ (colitis)	improve	-
2	M	58	Lung cancer	atezolizumab	2	same time	Skin	AIP-like	After discontinuation (1.5 months)	-	-	-	improve	-
3 [27]	F	70	Renal cancer	nivolumab	2	2 months	Endocrine (pituitaritis)	AIP-like	Discontinued	+ HC 10mg/day (pituitaritis)	same time	-	improve	-
4	M	71	Melanoma	nivolumab	2	2 weeks	Endocrine	Others (atrophy)	Discontinued	+ PSL 30mg/day	same time	-	improve	-
5 [28]	F	74	Renal cancer	nivolumab	5	2 days	Liver	AP-like (severe)	After discontinuation (3 months)	+ PSL 40mg/day	same time	+	die	-

ICI, immune checkpoint inhibitor; ICI-PI, ICI-related pancreatic injury; Grade, Grade of pancreatitis (Citation modified from CTCAE ver.5.0);

AIP, autoimmune pancreatitis; AP, acute pancreatitis; PSL, prednisolone; HC, Hydrocortisone
